# Frontal-to-Parietal Top-Down Causal Streams along the Dorsal Attention Network Exclusively Mediate Voluntary Orienting of Attention

**DOI:** 10.1371/journal.pone.0020079

**Published:** 2011-05-17

**Authors:** Takashi J. Ozaki

**Affiliations:** 1 Department of Life Sciences, University of Tokyo, Tokyo, Japan; 2 Laboratory for Dynamics of Emergent Intelligence, RIKEN Brain Science Institute, Wako, Saitama, Japan; Cuban Neuroscience Center, Cuba

## Abstract

Previous effective connectivity analyses of functional magnetic resonance imaging (fMRI) have revealed dynamic causal streams along the dorsal attention network (DAN) during voluntary attentional control in the human brain. During resting state, however, fMRI has shown that the DAN is also intrinsically configured by functional connectivity, even in the absence of explicit task demands, and that may conflict with effective connectivity studies. To resolve this contradiction, we performed an effective connectivity analysis based on partial Granger causality (pGC) on event-related fMRI data during Posner's cueing paradigm while optimizing experimental and imaging parameters for pGC analysis. Analysis by pGC can factor out exogenous or latent influences due to unmeasured variables. Typical regions along the DAN with greater activation during orienting than withholding of attention were selected as regions of interest (ROIs). pGC analysis on fMRI data from the ROIs showed that frontal-to-parietal top-down causal streams along the DAN appeared during (voluntary) orienting, but not during other, less-attentive and/or resting-like conditions. These results demonstrate that these causal streams along the DAN exclusively mediate voluntary covert orienting. These findings suggest that neural representations of attention in frontal regions are at the top of the hierarchy of the DAN for embodying voluntary attentional control.

## Introduction

Voluntary visual attentional control has been found to be mediated by large-scale distributed cortical regions across the frontal, parietal and visual cortices, called the dorsal attention network (DAN) [1]–[4]. This DAN model has integrated separate findings about the parietal [5]–[8] and frontal [9], [10] contributions to voluntary attentional control.

The DAN was first regarded as a parallel processing network, activated immediately upon the demands of voluntary attentional control [10]–[12]. This conventional concept has been challenged, however, since studies in primates have suggested causal relationships between the frontal and parietal regions, as revealed by multi-site single unit recordings [13] and microstimulation [14]. In humans, recent functional magnetic resonance imaging (fMRI) studies with effective connectivity analysis have suggested that voluntary attentional control is mediated by causal streams along the DAN from frontal to parietal or to the visual cortex [15], [16]. The combination of transcranial magnetic stimulation (TMS) and fMRI showed similar top-down frontoparietal causal streams during a visuospatial judgment task [17], [18], suggesting that the DAN is not a parallel but a serial processing network embodied by causal streams from frontal to parietal or to the visual cortex mediating voluntary attentional control.

Especially, our earlier study identified and quantified the difference in causal streams along the DAN between voluntary orienting of attention (orienting) and those for withholding attentional deployment (holding) [16]. This finding indicates that the DAN can flexibly change its network architecture on a basis of attentional states. Here we call it “dynamic networking” along the DAN.

However, some questions still remain, such as what happens to causal streams along the DAN when neither orienting nor holding occurs, and how causal streams vary without any explicit efforts in voluntary attentional control.

Resting-state fMRI has suggested that, during resting state, the DAN is configured by functional (not effective) connectivity without specifying directionality [19], [20]. This model has suggested that top-down causal streams along the DAN may also be configured, even during resting and other less-attentive states. That leads to an idea of “static networking” along the DAN, in contrast to our “dynamic networking” hypothesis. Thus, it is unclear whether the top-down causal streams along the DAN are or are not exclusively related to orienting. If so, then top-down causal streams along the DAN would occur even during resting state and only the strength of such causal streams would dissociate orienting from other less-attentional states.

To resolve this apparent contradiction between the “dynamic” and “static” networking hypothesis, we performed event-related fMRI experiments during Posner's cueing paradigm [21], followed by a comprehensive effective connectivity analysis with partial Granger causality (pGC), to quantify and evaluate causal streams along the DAN for orienting, holding, and other attentional states.

## Materials and Methods

### Participants

The study involved six healthy, neurologically normal male volunteers, all right-handed and ranging in age from 23–26 years, with normal or corrected-normal vision. All participants provided written informed consent, and the study protocol was approved by the Institutional Review Board of Ogawa Laboratories for Brain Function Research in accordance with the Declaration of Helsinki.

### Stimuli and procedure

**Figure 1:**
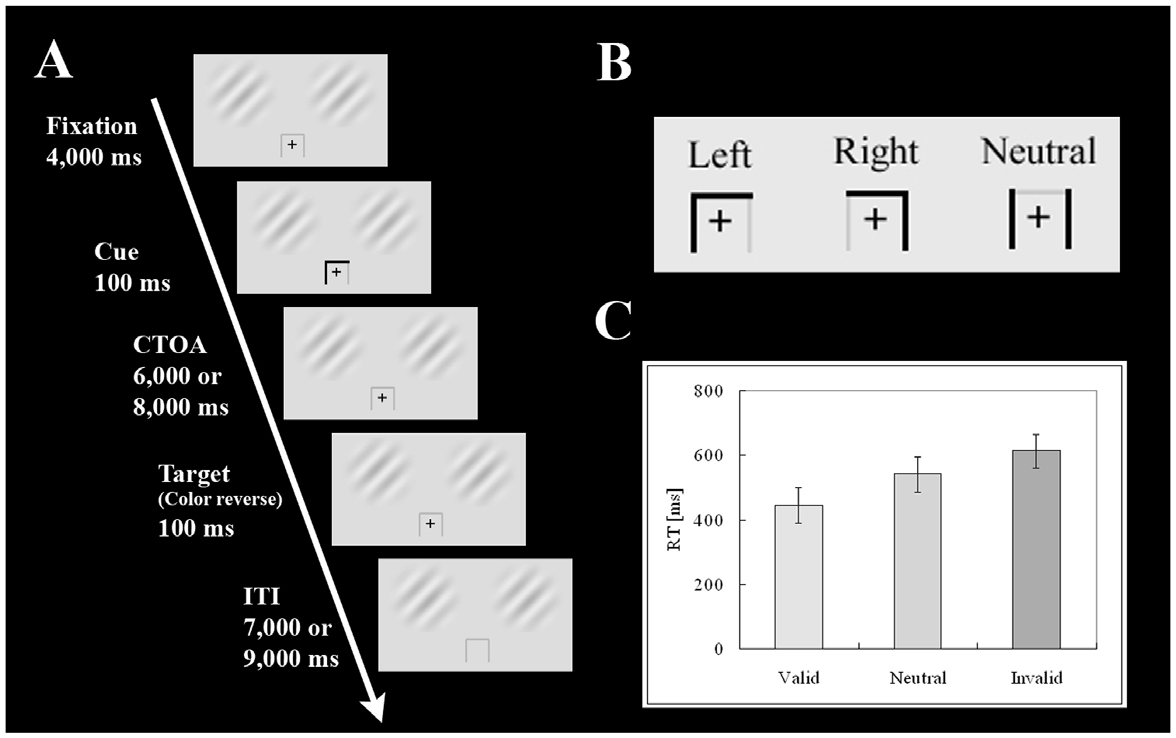
A summary of the experimental procedure. (**A**) Schematic representation of a procedure of the task. First, a cross was projected at the center of the visual field (duration 5,000 ms), followed by an arrow-shaped cue to indicate the direction to be paid attention (duration 100 ms). In this case, the participants had to pay attention to the left Gabor patch. After CTOA of 6,000 or 8,000 ms, a target appeared at either left or right position away from the center (duration 100 ms). The participants had to press a button if they successfully detected it. (B) Two types of cue; a “spatial cue” to the left or right, and a “neutral cue”. (C) Mean RT in each attentional condition (valid, neutral, and invalid). Error bars show SEM.

A gray cue template and two Gabor patches colored low-contrast green and red were projected onto a screen, which the participants viewed via a mirror mounted on the head coil of the MRI scanner. Throughout each session, the Gabor patches (diameter: 4.0°) were projected continuously in the right and left positions of the two upper quadrants of the visual field (Fig. 1A), with the center of each Gabor patch placed 4.0° from the fixation cross, and the cue template being 2.0°×2.0° in size (Fig. 1B).

At the beginning of each trial, a fixation cross was projected into the central visual field. This was followed 4,000 ms later by a “spatial cue”, consisting of a horizontal black line, with a descending vertical black line appearing either at its left or right end and indicating the left or right Gabor patch, respectively; or by a “neutral cue”, consisting of both the left and right descending vertical black lines without the horizontal line. The participants were instructed to attend to the cued location, i.e., the particular Gabor patch (spatial cue) or the fixation cross (neutral cue), while withholding manual responses and saccades (Fig. 1A, B). The cue disappeared 100 ms after onset and participants were asked to hold their attention on the cued location. After a period of 6,000 or 8,000 ms, assigned randomly to avoid anticipation as a cue-target onset asynchrony (CTOA), the color of one of the Gabor patches was reversed (red to green/green to red). The participants were instructed to press a button with their right index finger as soon as possible in response to this switch.

Trials were classified into four conditions: valid, invalid, neutral, and “null-cued”. In the valid condition, the Gabor patch indicated by the spatial cue reversed color (33.3% of trials). In the invalid condition, the Gabor patch that was not indicated by the spatial cue reversed color (8.3%). The cue validity was approximately 75%. In the neutral condition, participants were instructed to hold their attention on the fixation cross until one of the Gabor patches reversed color (16.7%). In the null-cued condition, no cue appeared prior to one of the Gabor patches reversing color, regardless of its location (16.7%). In the catch trials, neither of the Gabor patches reversed color following disappearance of the cue, and the participants had to withhold their response (25.0% for each condition except for the null-cued condition). In the null-cued condition, 66.6% of trials were catch trials in order to reduce the ability of participants to anticipate target appearance. Participants performed all trials in a random sequence (slow randomized event-related design). The two spatial cues and the neutral cue were presented randomly and with the same probability of occurrence. Based on the cost-benefit paradigm, the cost was defined as the response time (RT) in the invalid condition minus that in the neutral condition, and the benefit as neutral RT minus valid RT [22], [23].

To confirm stable fixation of each participant, electrooculography was recorded in a training session prior to an fMRI session and checked by visual inspection.

### fMRI data acquisition

fMRI data were acquired with a Magnetom Allegra 3.0 T MRI scanner system (Siemens, Erlangen, Germany). The functional volume was acquired for each participant with a T2*-weighted single-shot echo-planar imaging sequence (TR = 1,000 ms, TE = 30 ms, FoV = 224×224 mm, voxel size = 3.5×3.5×7.0 mm, 16 contiguous transverse slices, flip angle = 70°) sensitized to blood oxygenation level dependent (BOLD) contrast [24], [25]. TR≤1,000 ms was the optimal parameter for (partial) Granger causality analysis on event-related fMRI [26]. Each scan consisted of 72 runs, with each run consisting of 20 volumes. An anatomical volume was acquired for each participant using a Magnetization Prepared Rapid Acquisition Gradient Echo sequence (voxel size = 1.0×1.0×1.0 mm). Each anatomical volume was transformed into a standard stereotaxic atlas space based on Talairach coordinates [27].

### fMRI data analysis

fMRI data were analyzed and visualized using BrainVoyagerQX (Brain Innovation, Maastricht, The Netherlands). The first four volumes of each functional scan were discarded to allow stabilization of magnetization. After correction for slice scan time and head motion within a volume, functional volumes were coregistered with the Talairach space anatomical data sets to generate volume time courses. Each functional scan was high-pass filtered at 3 cycles per scan. Each voxel was spatially smoothed with a Gaussian filter of 7.0 mm full width at half maximum.

General linear models (GLM) were fitted to compute statistical parametric maps of the effects of the experimental conditions. The regressors were designed by calculating a square wave function, representing the event time course of the cues and targets, with a canonical hemodynamic response function (HRF). To detect orienting-related neural activation (linear contrast as [valid>neutral]) of the DAN [4], fixed effects analysis was performed in which the *P*-value threshold was set at *P*<0.05 (Bonferroni's correction, based on the volume of gray matter).

Regions of interest (ROIs) were determined from the obtained activation map based on (a) activation at a significance level *P*<0.0005 (corrected) and (b) volume size >50 mm^3^. Finally, 15 ROIs in a stereotaxic space were determined.

BrainVoyagerQX (Brain Innovation) was use to transform each representative anatomical volume into inflated and rendered three-dimensional images, on which computed group activation maps were overlaid.

### Basis of Granger causality analysis (GCA)

When one needs to elucidate any effective or causal connectivity from fMRI data, there are two major choices: one is Granger causality analysis (GCA) and/or its related methods [26], [28]–[30], and the other is dynamic causal modeling (DCM) [31]. As many previous studies or commentaries already argued [32]–[38], each method has advantages and limitations. In the current study, we chose GCA rather than DCM because a detail of visual inputs to regions along the DAN beyond visual cortices (in a bottom-up manner [39] or via a bypass [40]) is still unclear, while DCM requires an explicit input-output model [33], [37]. GCA can identify causal connectivity even without any explicit input-output model.

In general, Granger causality (GC) is tested on a basis of linear autoregressive models predicting the evolution of a time series or of a set of time series [29]. Univariate autoregressive models describe a single time series in terms of linear combinations of lags of the time-series. Furthermore, multivariate (vector) autoregressive (MVAR) models include lags of multiple time-series. To illustrate Granger causality, consider two time series X_1_(t) and X_2_(t) of length T. Suppose that the time evolutions of X_1_(t) and X_2_(t) can be described by a bivariate autoregressive model:
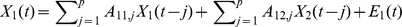


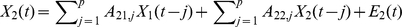
where p is the maximum number of lags included in the model (the model order, p<T), A_11/12/21/22_ contains the estimated coefficients of the model, and E_1_, E_2_ are residuals for each time series. If the variance of the prediction error E_1_ (or E_2_) is reduced by including the X_2_ (or X_1_) terms in the first (or second) equation, then it is said that X_2_ (or X_1_) Granger-causes X_1_ (or X_2_). In other words, X_2_ Granger-causes X_1_ if all the coefficients in A_12_ are jointly significantly different from zero. This can be tested by an F-test of the null hypothesis that A_12_ = 0, given assumptions of covariance stationarity on X_1_ and X_2_. The magnitude of a given Granger causality interaction can be estimated by the logarithm of the corresponding F-statistic [29].

### Partial Granger causality (pGC) analysis based on multivariate vector autoregressive (MVAR) model

To evaluate causal flows between ROIs, we computed pGC using Seth's Granger Causal Connectivity Analysis toolbox, based on multivariate vector autoregressive (MVAR) models including lags of multiple time-series [41], as described [42]. This type of causality analysis, based on MVAR models, can quantify and evaluate not only direct but also indirect causal connectivity [37], [38], while there are some controversies about its concept [36]. According to theoretical studies [29], MVAR models can reveal an independent causal index between time-series X_1_ and X_2_ even if the other variables, X_3_ … X_N_, mediate the causal flow between X_1_ and X_2_. That is, these MVAR models take all other variables (X_3_ … X_N_) into account and compute the causal index between X_1_ and X_2_ after considering the effects of all other variables (see [29]). Thus, the causality indices that we obtained effectively decreased any effects of possible mediators (X_3_ … X_N_).

In addition, pGC analysis was superior to conventional GC (e.g. conditional GC) analysis, in that it excluded exogenous or latent influences from unmeasured variables [32]. Because these influences are reflected by the correlations among the residuals of the regression, the analysis can factor out them by analogy with partial coherence [41], [42]. Therefore, the pGC indices that we computed show causal relationships only between pre-defined nodes.

pGC analysis and its statistical test was performed in two steps: individual level and subsequent group level, based on a method established in our earlier study [16]. This double-level analysis enables us to obtain inter-individually counterbalanced group causality indices with considering a large inter-individual variance of individual causality indices. At both levels, bootstrap methods were applied to evaluate empirical statistical significance [26]. Prior to analysis, the time course of averaged BOLD signals across all voxels in each ROI was extracted and normalized for each participant in order to avoid overestimations of causality [43].

On an individual level, sample F-values for each participant were first computed in both directions between the measured BOLD time series collapsed across trials of every ROI pair, in the orienting, holding, fixation, and ITI conditions. CTOA epochs (duration 6,000 or 8,000 ms) were analyzed for the orienting (spatial cue) and holding (neutral cue) conditions, whereas epochs for each condition were analyzed for the fixation (duration 4,000 ms) and ITI (duration 6,000 or 8,000 ms) conditions. Thus, each F-value indicates the probability that a BOLD time series of one ROI can explain the subsequent time series of the other ROI [26]. Second, to obtain an empirical null distribution, a bootstrap method was performed for each individual, in which 2,000 trial-randomized BOLD time series of each ROI were computed. Third, individual Z-values (Zi) for the group level analysis were computed by the rank-sum test, comparing the sample F-value with the empirical null distribution of F-values for each pair of ROIs and direction. The Zi obtained for each pair of ROIs and direction from each participant indicated the probability of causality in terms of statistics.

At the group level, a combined group Z-value (Zg) was first computed using the Stouffer method for each pair of ROIs and direction (adding all Zi for each pair of ROIs and direction and dividing the sum by the square root of the number of participants) [44]. Second, to estimate the empirical threshold for Zg, a group-level bootstrap method was performed, in which 2,000 bootstrap samples of Zg collapsed across ROIs and participants were computed as the empirical null distribution of Zg, with the empirical threshold (*P*<0.05) then determined as Zt. Finally, a causality index for each pair of ROIs and direction was computed as a simple sum of raw F-values across all participants when Zg was larger than Zt for each pair of ROIs. The resultant causality indices composed a 15×15 matrix (Fg matrix) for each experimental condition.

### Comparison of experimental conditions on causal streams

To identify the causal streams and evaluate which are more or less important for orienting at the group level and to compensate for the variability of HRF across cortical regions, we compared the group-level pGC indices across the four experimental conditions (orienting, holding, fixation, and ITI). Although HRF was highly variable across individuals and among different cortical regions of the same individual, HRF variations across cortical regions may not affect the comparison of conditions [41]. In contrast, we did not utilize the difference in influence term [26], [41], [45] to avoid underestimating pGC after comparisons across the four experimental conditions.

Each balanced causal stream (Fc) was computed for each experimental condition as Fc = X_0_−(X_1_+X_2_+X_3_)/3, in which X_0_ was one condition of interest and X_1…3_ were the others. Each Fc was normalized by the maximum F-value in each 15×15 matrix and F-values less than zero were set to 0 because non-positive F-values were regarded as non-causal. Finally, each Fc (15×15 matrix) was used to describe causal streams among the ROIs for each of the four experimental conditions.

For visualization, the causal streams were drawn on both a 2D-graph and a 3D-rendered and transparent cortex image with 3D-rendered images of the ROIs. In both the graphs and the 3D cortex images, the causal streams were represented by green arrows and the strength of each stream (normalized to 1) was represented by the thickness of the arrow.

## Results

We first performed an event-related fMRI experiment using the Posner cueing paradigm [21], after optimizing experimental and imaging parameters for partial Granger causality (pGC) analysis (see Methods and Materials). This was followed by a pGC analysis on the fMRI data, to quantify and evaluate causal streams along the DAN for orienting, holding, and other attentional states. By analogy with partial coherence, pGC analysis can factor out exogenous and latent influences better than conditional GC (cGC) analysis, therefore the pGC analysis results show only the causal relationships among the pre-defined ROIs. Finally, we tested our hypothesis by comparing causal streams across the four experimental conditions (orienting, holding, fixation, inter-trial interval/ITI; see Methods and Materials).

### Behavior

Electrooculography recorded in a training session prior to an fMRI session confirmed stable fixation of each participant (data not shown). Figure 1C shows that the mean ± SEM response times (RT) of the participants in the imaging sessions under valid, neutral, and invalid conditions were 444±54 ms, 541±55 ms, and 613±51 ms, respectively. Statistical analyses indicated showed that attention was associated with significant effects on costs (t_5_ = 2.73, *P*<0.05) and benefits (t_5_ = 3.54; *P*<0.05).

### Event-related fMRI

**Figure 2:**
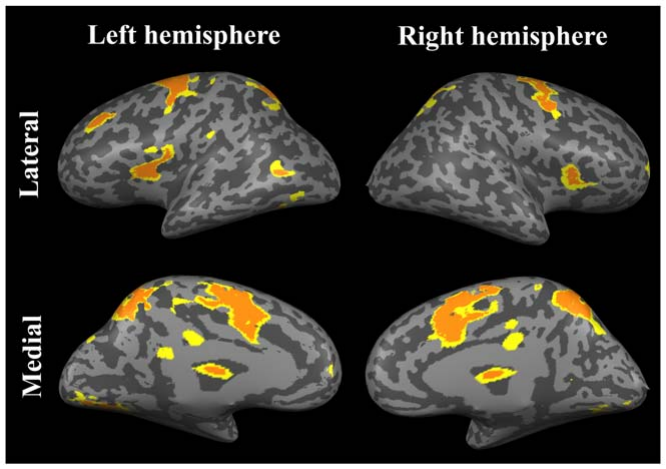
Significant activation of typical regions along the DAN and other regions during orienting more than during holding of attention with a linear contrast as [valid>neutral] (*P*<0.05, Bonferroni's correction, yellow and orange regions). Orange regions were selected as ROIs.

A general linear model analysis of the event-related fMRI data showed that, consistent with previous findings [12], [46], typical regions along the DAN and other regions were significantly more activated during orienting than during holding of attention, with a linear contrast as [valid>neutral] (*P*<0.05, Bonferroni's correction; Figure 2, yellow and orange regions). In contrast, no regions were significantly activated with an opposite contrast (i.e. more during holding than during orienting, as [neutral>valid]).

### pGC analysis

Among all activated clusters, 15 regions were selected as ROIs (Figure 2, orange regions only) and causal streams indexed by pGCs among these ROIs were computed (see Materials and Methods). The ROIs included the human frontal eye field (hFEF), the posterior parietal cortex (PPC), the medial frontal cortex (mFC), a complex of the inferior frontal gyrus and the anterior insular cortex (IFG-AIC), the middle frontal gyrus (MFG), a complex of the frontal operculum and the anterior insular cortex (FO-AIC), and the lateral occipital cortex (LOC). “R” and “L” indicate the right and left hemispheres, respectively (See Table S1 for details on the ROIs).

**Figure 3:**
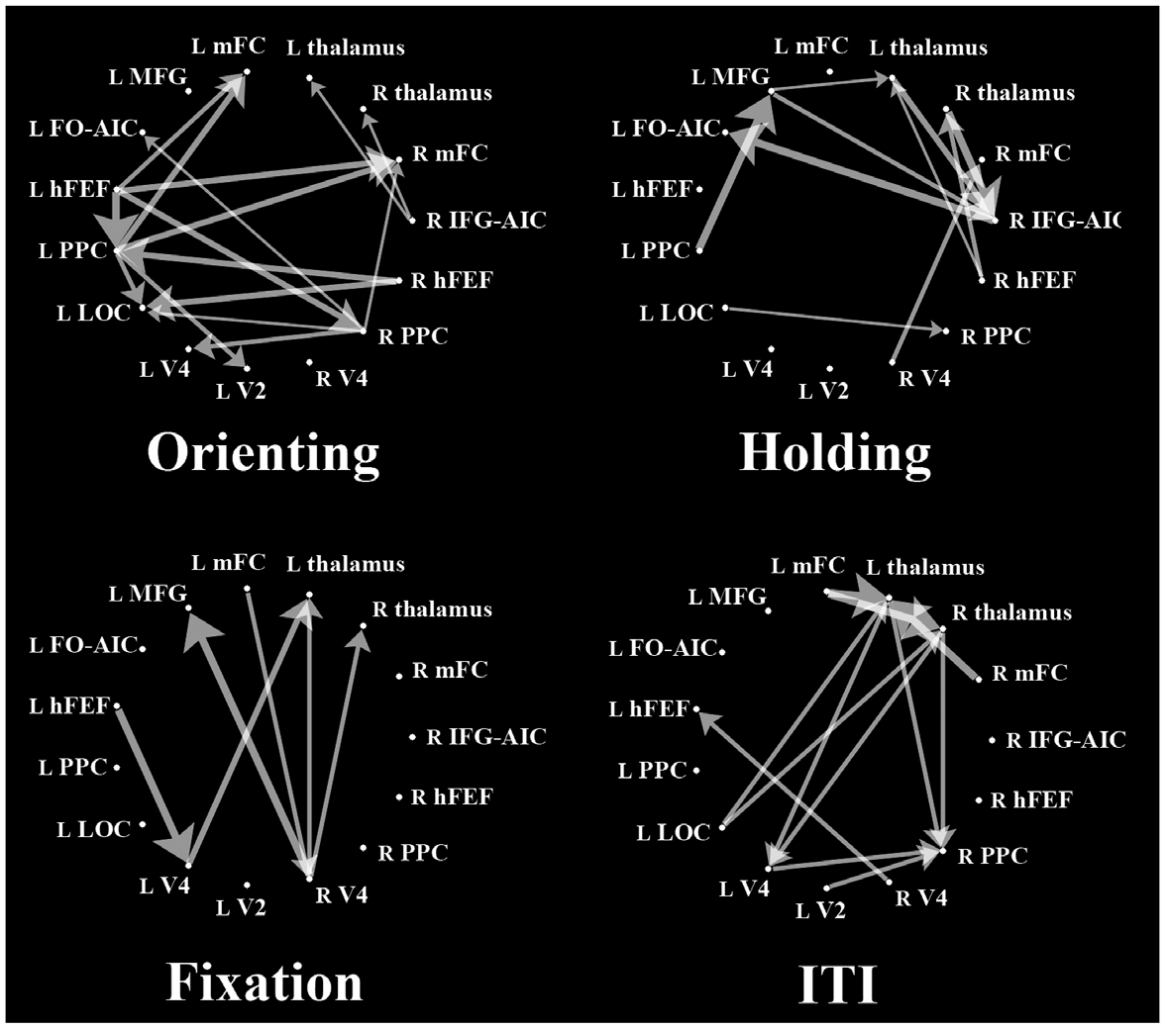
Network graphs in the four experimental conditions obtained from pGC analysis. Unidirectional arrows indicate directional causal streams from one ROI to another. (ROI abbreviations: R, right; L, left; hFEF, human frontal eye field; PPC, posterior parietal cortex; mFC, medial frontal cortex; IFG-AIC, inferior frontal gyrus-anterior insular cortex; MFG, middle frontal gyrus, FO-AIC, frontal operculum-anterior insular cortex; LOC, lateral occipital cortex. See Table S1 for details of the ROIs.

**Figure 4:**
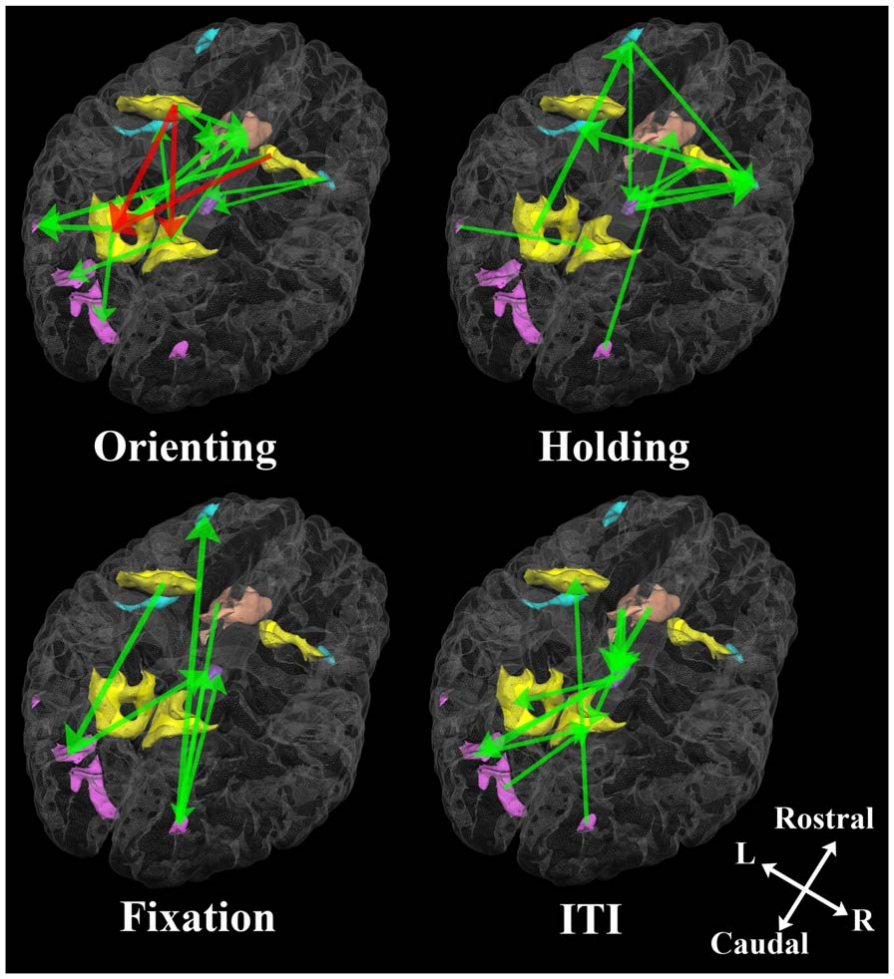
Network graphs of pGC in the four experimental conditions shown in **Figure 3** overlaid onto a transparent and 3D-rendered gray matter volume. Green unidirectional arrows indicate statistically significant causal streams from one ROI to another. Red unidirectional arrows mean significant frontal-to-parietal causal streams along the DAN. Regions along the DAN are shown in yellow, the visual cortices are in pink, the medial frontal cortex is in salmon pink, the thalami in purple and the other regions in blue.

At the group level, Figure 3 shows four graphs describing causal streams indexed by balanced pGC values among the ROIs of the four experimental conditions (orienting, holding, fixation, and ITI). Unidirectional arrows indicate causal streams from one ROI to another. For visualization, Figure 4 shows the same graphs overlaid onto 3D-rendered transparent cortex images. Each graph shows the results of comparisons of each experimental condition. Red unidirectional arrows represent frontal-to-parietal top-down causal streams along the DAN (hFEF→PPC), whereas green arrows represents causal streams not in the top-down direction.

For orienting, some frontal-to-parietal top-down causal streams along the DAN appeared; the L hFEF sent causal streams to the L and R PPC, and the R hFEF sent causal streams to the L PPC and LOC. The L PPC sent streams to the L V2 and LOC, and the R PPC sent a stream to the L V4 as an extension of the DAN. During holding, there were few or no top-down causal streams along the DAN; rather, they converged on the R IFG-AIC from various ROIs outside the DAN. Our results for orienting indicate that our pGC analysis successfully replicated previous findings [16], although our results for holding may update previous findings.

Under conditions other than orienting or holding, there were no systematic causal streams along the DAN, although one causal stream along the DAN, from the frontal to the visual cortex, was detected during fixation.

On the other hand, causal streams from the frontal to visual regions (hFEF/mFC→V2/V4/LOC), which have been investigated by neurophysiological [13], [47], [48] or human imaging studies [15], [49], appeared during orienting and fixation (Figure 4). This finding indicates that such top-down streams from the frontal to visual regions are not exclusive streams for orienting, in contrast to streams from the frontal to parietal regions.

To summarize comparisons across the four experimental conditions, we observed frontal-to-parietal top-down causal streams along the DAN only for orienting, indicating that these causal streams occur exclusively during orienting, but not during other conditions. All causality indices across the 15 ROIs for the four experimental conditions are shown in Tables S2, S3, S4, and S5.

## Discussion

Our fMRI data and the results of pGC analysis indicate that frontal-to-parietal top-down causal streams along the DAN were exclusively related to voluntary orienting of attention (orienting), not to any other less-attentive states. This finding provides an important update of our earlier study [16]; frontal-to-parietal top-down causal streams are not only more dominant during orienting than during holding, but also exclusive during orienting in comparison with any other less-attentive states including holding. This confims our “dynamic networking” hypothesis, rather than “static networking”.

### pGC analysis: orienting

Although our pGC analysis could not replicate all of the causal streams between other ROIs observed during our previous GC analysis [16], our findings indicate the robustness of these frontal-to-parietal top-down causal streams along the DAN for orienting, regardless of analysis by cGC or pGC.

In contrast to the conventional view, that the DAN as an attention-controlling circuit should process only contralateral orienting in a hemisphere-symmetric manner [50], our findings indicate a hemispherical asymmetry in the top-down causal streams along the DAN for orienting. However, our findings may be plausible because hemispherical asymmetry has been observed in the DAN and in oculomotor functional structures [51], [52].

### pGC analysis: holding, fixation and ITI as less-attentive states

Our pGC results update our earlier cGC findings [16], because unlike cGC analysis, pGC analysis can factor out exogenous and/or latent influences from causality indices. Thus, our results represent purely causal relationships between pre-defined ROIs. The results presented here indicate that the R IFG-AIC may be important for holding as an update of our earlier study [16].

Unlike orienting or holding, no systematic causal streams along the DAN appeared under fixation and ITI conditions. This confirms our working hypothesis that frontal-to-parietal top-down causal streams along the DAN are exclusively mediated by orienting and they do not occur during less-attentive states. Surprisingly, only a few studies have assessed effective connectivity during such less-attentive states including the resting state. For example, one study reported effective connectivity across eight RSNs, as determined by independent component analysis using cGC, but did not assess effective connectivity across focused neural regions [53]. Future studies are required to elucidate causal streams along the DAN during less-attentive states, such as fixation and ITI.

Our results also show that frontal-to-parietal top-down causal streams revealed by effective connectivity (pGC) analysis along the DAN occur exclusively during voluntary covert orienting but not during less-attentive states, although previous functional connectivity studies have indicated that the DAN is configured in the resting-state as its less-attentive state.

### hFEF→PPC vs. hFEF→visual cortex

Our current results propose that causal streams from the hFEF to the PPC are the most important neural components for voluntary attentional control. On the other hand, the results also suggest that streams from the frontal to visual regions may not be exclusive for orienting and they also appear during fixation, in contrast to those from the frontal to parietal regions.

Indeed, top-down flows from the frontal to visual regions have been examined in recent neurophysiological [13], [47] or human imaging studies [15] based on anatomical findings of fiber tracts between the frontal and visual regions [54]. Microstimulation studies also proposed that microstimulation to the FEF in non-human primates could manipulate both attentive behavior and V4 neuronal activity [14], [55], [56]. This line of evidence suggests that information flow from the frontal to visual regions is important for voluntary attentional control as well as those from the frontal to parietal regions.

This discrepancy between our results and the previous findings can be explained as follows: shortly, causal streams from the frontal to parietal (not visual) regions have attracted less attention, especially in the non-human primate studies. That is why there have been almost no reports of frontal-to-parietal causal streams in non-human primate studies. From this viewpoint, our current results appear as novel evidence of frontal-to-parietal causal streams that have been neglected in many previous studies.

The reason that causal streams from the frontal to visual regions appeared not only for orienting but also for fixation is less clear. However, it can be explained by a task structure. In the current task, the fixation cross always appears prior to the spatial (orienting) or neutral (holding) cue; that can facilitate some attentive states at a center of the visual field where subsequent cues appear. Indeed, it has been known that the fixation cross or point itself can affect attentive states to some degree [23]. Such a task structure may cause confounded effects and it should be improved in future studies.

However, this process does not include any aspects of voluntary attentional control of orienting, and it is consistent with the current finding that the fixation epoch did not yield any frontal-to-parietal causal streams. That also confirms the finding that frontal-to-parietal top-down causal streams exclusively appeared during voluntary attentional control of orienting.

### Hierarchy of neural representation of visual attention in the DAN

Our results also suggest that the frontal regions in the DAN may have priority over the other regions in their neural representation of visual attention. Our pGC analysis showed that the hFEF is superior to the PPC and other visual regions in causal relationships.

Although the neural origin of visual attention is less clear, many neurophysiologic and functional neuroimaging imaging studies have assessed the neural representation of visual attention in the visual [48], [49], parietal [5]–[7], [57], [58] and frontal [2], [9], [13], [58]–[62] regions along the DAN. These findings have indicated that each subregion of the DAN has common or similar neural representation of visual attention. However, the region at the top of the DAN hierarchy, as described by the neural representation of visual attention, has not yet been determined. However, our pGC analysis of event-related fMRI data during voluntary attentional control suggests that the hFEF is a strong candidate for the region at the top of the DAN hierarchy, in agreement with previous findings in neuroscience. Indeed, monkey FEF (mFEF) has been shown to receive earlier visual input from the superior colliculus (SC) [39], [40], [63], and neuropsychological studies with blind human patients have indicated that this model can be applied to the human brain [64], [65]. Importantly, “bypass” visual inputs via the SC reach the mFEF earlier than regions along the DAN that receive inputs via the lateral geniculate nucleus (LGN) [39]. These findings provide further evidence that the m/hFEF, which receives bypass visual inputs, is superior to other regions along the DAN in voluntary attentional control based on visual information.

Several studies on the neural substrates of saccadic eye movement have shown that the m/hFEF has network superiority over the PPC. In non-human primates, the dorsal premotor cortex reorganizes information processed in the PPC [66]. In humans, however, the hFEF is critically involved in the preparatory set while the PPC is involved in the execution of saccades [67]. These findings thus support the hypothesis, that the m/hFEF is at the top of the hierarchy of neural representation of visual attention, thus explaining some phenomena related to visual attention. For example, the premotor theory of attention can be explained by the inclusion of the m/hFEF in the premotor cortex because the m/hFEF, which controls visual attention, may also affect motor programming in the premotor cortex [68].

Our conclusion, that the m/hFEF is at the top of the hierarchy of neural representation of visual attention, leads to some novel questions and future directions. For example, it is unclear whether the neural representation of visual attention is or is not shared among all regions along the DAN, though that question has been investigated in the frontal [9], [14], [61] and parietal [5], [57], [58], [61] cortices. A resolution of this question is needed to understand the entire attention controlling system in the human brain as a behaviorally-decision making system [69].

To summarize, using a novel pGC analysis on event-related fMRI data during Posner's paradigm and optimizing experimental and imaging parameters for pGC analysis, we confirmed the hypothesis, that frontal-to-parietal top-down causal streams along the DAN exclusively mediate voluntary orienting of attention. Our results also showed that less systematic causal streams along the DAN and across other attention-related regions are involved in less-attentive states including holding. These findings further suggest that the hFEF is an origin of the frontal-to-parietal top-down causal streams along the DAN and that the hFEF should be at the top of the hierarchy of neural representation of visual attention.

## Supporting Information

Table S1 Details of all ROIs of activation for pGC analysis.
(XLS)

Table S2 pGC indices across the 15 ROIs under the orienting condition.
(XLS)

Table S3 pGC indices across the 15 ROIs under the holding condition.
(XLS)

Table S4 pGC indices across the 15 ROIs under the fixation condition.
(XLS)

Table S5 pGC indices across the 15 ROIs under the ITI condition.
(XLS)
